# Genome sequence and description of *Corynebacterium ihumii* sp. nov.

**DOI:** 10.4056/sigs.5149006

**Published:** 2014-03-15

**Authors:** Roshan Padmanabhan, Grégory Dubourg, Jean-Christophe Lagier, Carine Couderc, Caroline Michelle, Didier Raoult, Pierre-Edouard Fournier

**Affiliations:** 1 URMITE, UM63, CNRS7278, IRD198, Inserm1095, IHU Méditerranée-Infection, Aix-Marseille Université, Faculté de médecine, France; 2 Special Unit Agents, King Fahd Medical Research Center, King Abdul Aziz University, Jeddah, Saudi Arabia

**Keywords:** *Corynebacterium ihumii*, genome, culturomics, taxono-genomics

## Abstract

*Corynebacterium ihumii* strain GD7^T^ sp. nov. is proposed as the type strain of a new species, which belongs to the family *Corynebacteriaceae* of the class *Actinobacteria*. This strain was isolated from the fecal flora of a 62 year-old male patient, as a part of the culturomics study. *Corynebacterium ihumii* is a Gram positive, facultativly anaerobic, nonsporulating bacillus. Here, we describe the features of this organism, together with the high quality draft genome sequence, annotation and the comparison with other member of the genus *Corynebacteria*. *C. ihumii* genome is 2,232,265 bp long (one chromosome but no plasmid) containing 2,125 protein-coding and 53 RNA genes, including 4 rRNA genes. The whole-genome shotgun sequence of *Corynebacterium ihumii* strain GD7^T^ sp. nov has been deposited in EMBL under accession number GCA_000403725.

## Introduction

*Corynebacterium ihumii* strain GD7^T^ sp. nov. (= CSUR P902, = DSM 45751) is the type strain of *Corynebacterium ihumii* strain GD7^T^ sp. nov. This bacterium is a Gram-positive, facultativly anaerobic, non spore-forming, non-motile bacillus that was isolated from the stool of a 62 year-old French male who was admitted to the intensive care unit in the Timone Hospital, Marseille, France, for respiratory distress. This strain was isolated as a part of “culturomics” project whose scope is to cultivate all species within human feces [1,2].

The current classification of prokaryotes is based on a combination of phenotypic and genotypic characteristics [3,4] that include 16S rRNA gene phylogeny and nucleotide sequence similarity, G + C content and DNA–DNA hybridization (DDH). Despite being considered as a “gold standard” these genotypic tools exhibit several drawbacks that are overcome by newer sequencing methods [5,6]. Because of the rapidly declining cost of sequencing, the number of sequenced bacterial genomes rapidly increased (almost 7,000 to date [7]). Hence, we recently proposed to incorporate genomic information among criteria used for the description of new bacterial species [8-29].

*Corynebacteria* are Gram-positive bacteria that belong to the phylum *Actinobacteria* and have a high G+C content. They are found in diverse ecological niches such as soil, clinical specimens, cheese smear, vegetables, sewage etc. The genus *Corynebacterium* was created by Lehmann and Neumann in 1896 [30] which currently comprises 112 distinct species and 11 subspecies [31]. Many *Corynebacterium* species are involved in human and animal diseases and include *C. diphtheriae* [32], *C. jeikeium*, *C. urealyticum*, *C. striatum, C. pseudotuberculosis, and C. ulcerans* [33]. Others have industrial applications for amino acid production like *C. glutamicum* [34].

Here, we present a summary classification and a set of features for *Corynebacterium ihumii* strain GD7^T^ sp. nov. (=CSUR P902, =DSM 45751) together with the description of the genome sequencing and annotation.

## Classification and Features

A stool sample was collected from a 62 year-old male admitted to the intensive care unit of the Timone Hospital in Marseille, France. The patient gave a written informed consent for the study. The study was approved by the Ethics Committee of the Institut Fédératif de Recherche IFR48, Faculty of Medicine, Marseille, France, under agreement number 09-022. The fecal specimen was preserved at -80°C after collection. Strain GD7^T^ (Table 1) was isolated in January 2012 by cultivation on PVX agar (BioMerieux, Marcy l’Etoile, France) in aerobic condition with 5% CO_2_ at 37°C, after 21 days of incubation.

**Table 1:** Classification and general features of *Corynebacterium ihumii* strain GD7^T^ according to the MIGS recommendations [35]

**MIGS ID**	**Property**	**Term**	**Evidence codes^a^**
	Current classification	Domain *Bacteria*	TAS [36]
		Phylum *Actinobacteria*	TAS [37]
		Class *Actinobacteria*	TAS [38]
		Order *Actinomycetales*	TAS [38-41]
		Family *Corynebacteriaceae*	TAS [38-40,42]
		Genus *Corynebacterium*	TAS [39,43,44]
		Species *Corynebacterium ihumii*	IDA
		Type strain GD7	IDA
	Gram stain	positive	IDA
	Cell shape	rod	IDA
	Motility	non motile	IDA
	Sporulation	non endospore forming	IDA
	Temperature range	mesophilic	IDA
	Optimum temperature	37°C	IDA
MIGS-6.3	Salinity	unknown	IDA
MIGS-22	Oxygen requirement	facultative anaerobic	IDA
	Carbon source	unknown	NAS
	Energy source	unknown	NAS
MIGS-6	Habitat	human gut	IDA
MIGS-15	Biotic relationship	free living	IDA
MIGS-14	Pathogenicity Biosafety level Isolation	unknown 2 human feces	IDA
MIGS-4	Geographic location	France	IDA
MIGS-5	Sample collection time	January 2012	IDA
MIGS-4.1	Latitude	43.296482	IDA
MIGS-4.1	Longitude	5.36978	IDA
MIGS-4.3	Depth	Surface	IDA
MIGS-4.4	Altitude	0 m above sea level	IDA

^a^ Evidence codes - IDA: Inferred from Direct Assay; TAS: Traceable Author Statement (i.e., a direct report exists in the literature); NAS: Non-traceable Author Statement (i.e., not directly observed for the living, isolated sample, but based on a generally accepted property for the species, or anecdotal evidence). These evidence codes are from the Gene Ontology project [45]. If the evidence is IDA, then the property was directly observed for a live isolate by one of the authors or an expert mentioned in the acknowledgements.

**Figure 1:**
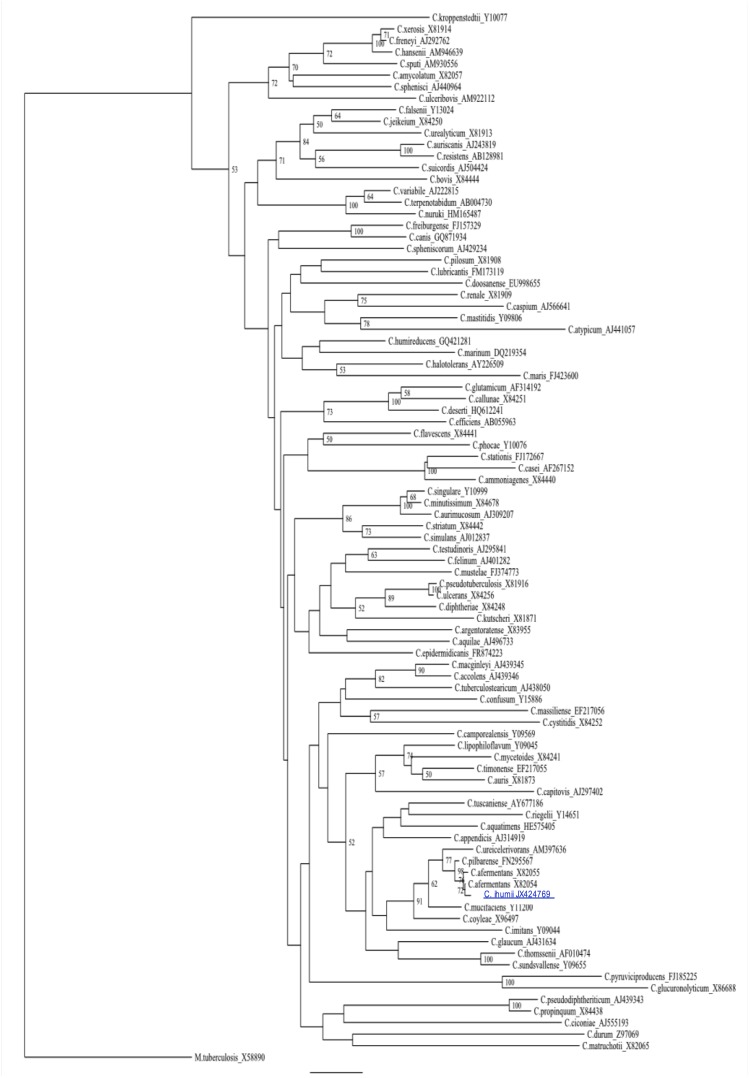
Phylogenetic tree highlighting the position of *Corynebacterium ihumii* strain GD7^T^ relative to other type strains within the *Corynebacterium* genus. GenBank accession numbers are indicated for each strain. Sequences were aligned using CLUSTALW, and phylogenetic inferences obtained using the neighbor-joining method within the MEGA software. Numbers at the nodes are percentages of bootstrap values obtained by repeating the analysis 1,000 times to generate a majority consensus tree. *Mycobacterium tuberculosis* was used as an outgroup. The scale bar represents a 2% nucleotide sequence divergence.

To understand the phylogenetic relationships of *C. ihumii* GD7^T^, we constructed a 16S rRNA-based neighbor joining tree with 90 *Corynebacterium* species (Figure 1). The 16S rRNA sequence similarity among *Corynebacterium* species ranged from 82.9 to 99.60%. Strain GD7^T^ exhibited a highest 16S rRNA sequence similarity of 99.1% with *C. pilbarense*. This value, although higher than the 98.7% 16S rRNA gene sequence threshold recommended by Stackebrandt and Ebers to delineate a new species without carrying out DNA-DNA hybridization [4], is in the range of values observed within the *Corynebacterium* genus.

**Figure 2:**
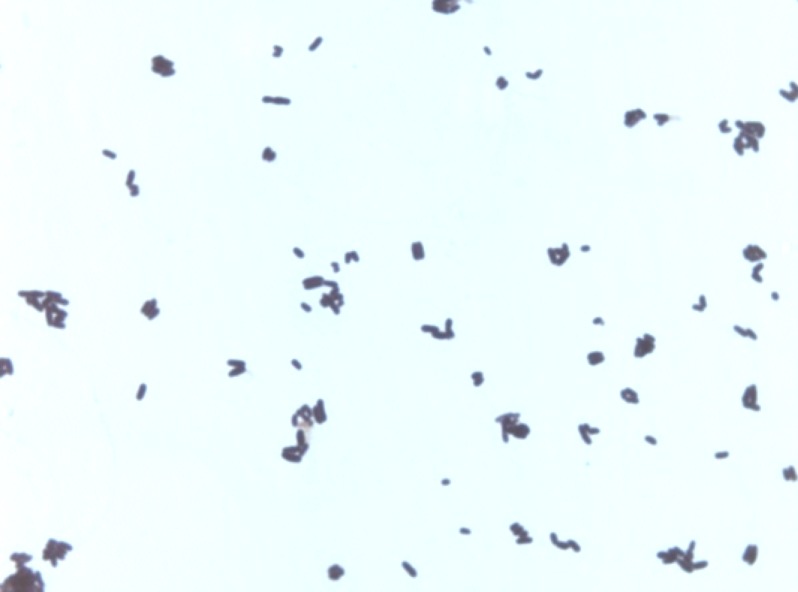
Gram staining of *C. ihumii* strain GD7^T^

**Figure 3:**
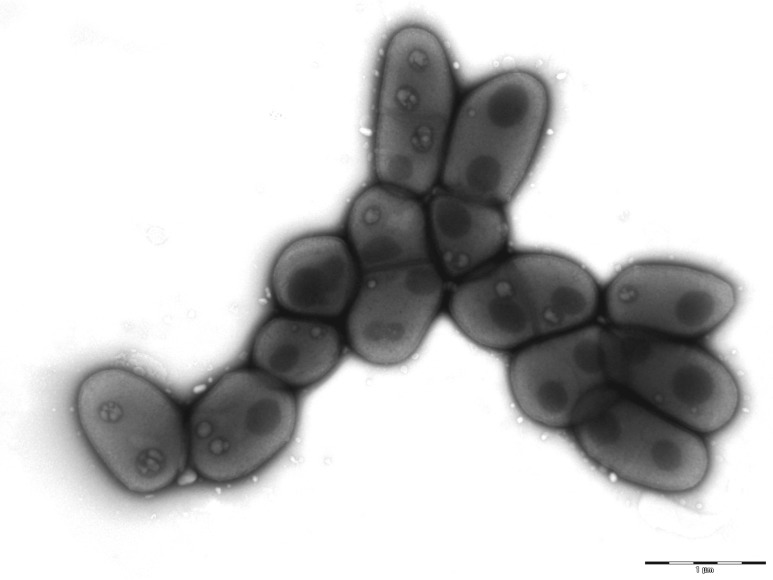
Transmission electron microscopy of *C. ihumii* strain GD7^T^, using a Morgani 268D (Philips) at an operating voltage of 60kV. The scale bar represents 1 μm.

Various growth temperatures (25, 30, 37, 45 and 56°C) were tested. Growth occurred between 30 and 45°C on blood-enriched Columbia agar (BioMérieux), with the optimal growth being obtained at 37°C. Growth of the strain was tested under anaerobic and microaerophilic conditions using the GENbag Anaer and GENbag microaer systems, respectively (BioMérieux), and under aerobic conditions, with or without 5% CO_2_. Optimal growth was achieved aerobically, but cell growth was also observed under microaerophilic and anaerobic conditions. The motility test was negative and the cells were nonsporulating. Colonies were white and granular with a diameter of 0.5 mm on blood-enriched Columbia agar (BioMérieux). Gram staining showed short Gram-positive rods (Figure 2). By electron microscopy, cells grown on agar had a mean length and diameter of 1.26 µm (range 1.1 – 1.4) and 0.7 µm (range 0.6-0.85), respectively (Figure 3).

Strain GD7^T^ was catalase positive and oxidase negative. Using the API ZYM system (BioMérieux), positive reactions were observed for alkaline phosphatase, leucine arylamidase, valine arylamidase, cystine arylamidase, acid phosphatase and naphthol-AS-BI-phosphohydrolase. Negative reactions were observed for esterase (C4), esterase lipase (C8), lipase (C14), trypsin, α-chemotrypsin, α-galactosidase, β-galactosidase, β-glucuronidase, α-glucosidase, N actetyl-β-glucosaminidase, α-mannosidase and α-fucosidase. Using the API CORYNE system (BioMérieux), positive reactions were observed for pyrazinamidase, alkaline phosphatase, and glucose and ribose fermentation. Negative reactions were observed for reduction of nitrates, pyrolidonyl arylamidase; β-glucuronidase, β-galactosidase, α-glucosidase N-acetyl-β-glucosaminidase, β-glucosidase, urease, gelatin hydrolysis, fermentation of xylose, mannitol, maltose, lactose, saccharose and glycogen. Using an API 50CH strip (BioMérieux), positive reactions were observed for fermentation of L-arabinose, D-ribose, D-xylose, methyl-βD xylopranoside, D-galactose, D-glucose, D-fructose, D-mannose, L-rhamnose, D-mannitol, methyl-αD-xylopranoside, methyl-αD-glucopranoside, N-acetylglucosamine, amygdalin, arbutin, salicin, D-cellobiose, D-maltose, D-lactose, D-mellibiose, D-saccharose, D-trehalose, inulin, D-raffinose, amidon, glycogen and D-lyxose. Negative reactions were observed for fermentation of glycerol, erythritol, D-arabinose, L-xylose, D-adonitol, L-sorbose, dulcitol, inositol, D-sorbitol, esculin ferric citrate, D-melezitose, D- xylitol, gentiobiose, D-turanose, D-tagatose, D-fucose, L-fucose, D-arabitol, L-arabitol, potassium gluconate, and potassium 2-ketogluconate. Table 2 summarizes the differential phenotypic characteristics of *C. ihumii*, *C. pilbarense*, *C. coylae*, *C. glaucum*, and *C. mucifaciens*. *C. ihumii* strain GD7^T^ was susceptible to amoxicillin, amoxicillin-clavulanic acid, ceftriaxone, imipenem, doxycycline, vancomycin, erythromycin, rifampicin, trimethoprim/sulfamethoxazole and ciprofloxacine whereas it was resistant to metronidazole.

**Table 2:** Differential characteristics of *C. ihumii* sp. nov. strain GD7^T^, *C. pilbarense, C. coylae, C. glaucum* and *C. mucifaciens*.

**Properties**	*C. ihumii*	*C. pilbarense*	*C. coylae*	*C. glaucum*	*C. mucifaciens*
Colony size (mm)	0.5	0.5 – 2.0	1.0	na	1.0 – 1.5
Oxygen requirement	facultative anaerobic	facultative anaerobic	facultative anaerobic	facultative anaerobic	facultative anaerobic
Gram stain	+	+	+	+	+
Motility	-	-	-	-	-
Endospore formation	-	-	-	-	-
**Production of**					
Alkaline phosphatase	+	+	+	+	+
Acid phosphatase	+	+	+	-	+
Catalase	+	+	+	+	+
Oxidase	-	-	-	-	-
Nitrate reductase	-	-	-	-	-
Urease	-	-	-	-	-
α-galactosidase	-	-	-	-	-
β-galactosidase	-	-	-	-	-
β-glucuronidase	-	-	-	-	-
α -glucosidase	-	-	-	-	-
β-glucosidase	-	-	-	-	-
Esterase	-	-	+	-	+
Esterase lipase	-	-	+	+	+
naphthol-AS-BI-phosphohydrolase	+	+	na	+	na
N-acetyl-β-glucosaminidase	-	-	-	-	-
Pyrazinamidase	+	+	+	+	+
α-mannosidase	-	-	-	-	-
α-fucosidase	-	-	-	-	-
Leucine arylamidase	+	+	+	+	na
Valine arylamidase	+	-	-	-	-
Cystine arylamidase	-	-	+	-	+
α-chemotrypsin	-	-	-	-	-
Trypsin	-	-	-	-	-
**Utilization of**					
5-keto-gluconate	-	na	+	na	-
D-xylose	+	-	-	-	-
D-fructose	+	na	+	na	+
D-glucose	+	+	+	+	+
D-mannose	+	na	+	na	+
Habitat	Human gut	Human joint fluid	Human blood	Cosmetic dye	Human blood

na = data not available

**Figure 4:**
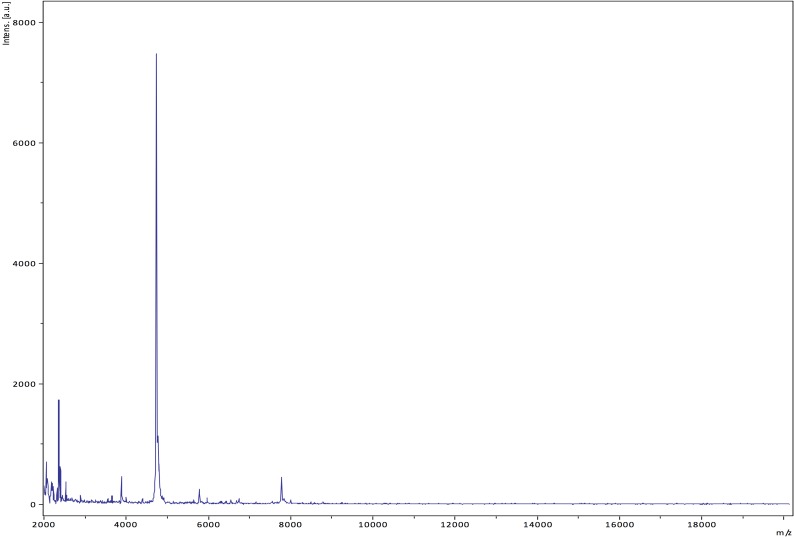
Reference mass spectrum from *C. ihumii* strain GD7^T^. Spectra from 12 individual colonies were compared and a reference spectrum was generated.

**Figure 5:**
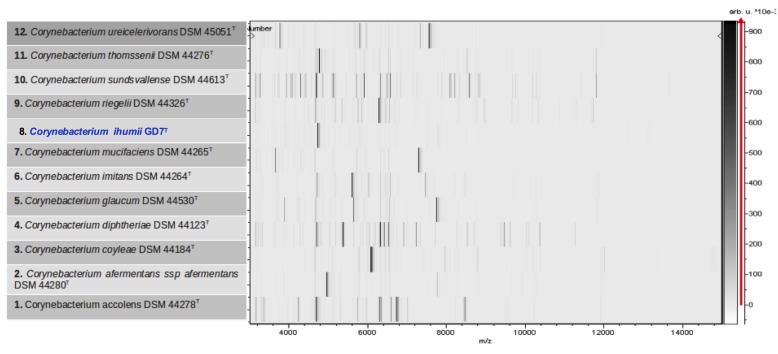
Gel view comparing *C. ihumii* sp. nov. strain GD7^T^ (= CSUR P902 = DSM 45751) to other members of the *Corynebacterium* genus. The gel view displays the raw spectra of all loaded spectrum files arranged in a pseudo-gel like look. The x-axis records the m/z value. The left y-axis displays the running spectrum number originating from subsequent spectra loading. The peak intensity is expressed by a Gray scale scheme code. The color bar and the right y-axis indicate the relation between the color a peak is displayed with and the peak intensity in arbitrary units.

Matrix-assisted laser-desorption/ionization time-of-flight (MALDI-TOF) MS protein analysis was peformed as previously described [46] using a Microflex spectrometer (Bruker Daltonics, Leipzig, Germany). The spectra from twelve isolated distinct GD7^T^ colonies were imported into the MALDI BioTyper software (version 2.0, Bruker) and analyzed by standard pattern matching (with default parameter settings) against the main spectra of 4,706 bacteria, including spectra from validated *Corynebacterium* species, that were part of the reference data contained in the BioTyper database. The presumptive identification and discrimination of the tested species from those in the database was interpreted as follows: a score > 2 with a validly published species enabled the identification at the species level; a score > 1.7 but < 2 enabled the identification at the genus level; and a score < 1.7 did not enable any identification. For strain GD7^T^, no significant score was obtained, suggesting that GD7 isolate was not a member of any known species or genus (Figures 4 and 5).

## Genome sequencing information

### Genome project history

As part of a 'culturomics' study of the human digestive flora, this organism was isolated and selected for sequencing on the basis of its phenotypic differences, phylogenetic position and 16S rRNA and *rpoB* sequence similarity to other members of the genus *Corynebacterium* [1,2]. It is the first sequenced genome of *C. ihumii* sp. nov. The GenBank Bioproject number is PRJEB646 and consists of 41 large contigs in 5 scaffolds. Table 3 shows the project information and its association with MIGS version 2.0 compliance [47].

**Table 3:** Project information

**MIGS ID**	**Property**	**Term**
MIGS-31	Finishing quality	High-quality draft
MIGS-28	Libraries used	One 454 paired end 3-kb library
MIGS-29	Sequencing platforms	454 GS FLX Titanium
MIGS-31.2	Fold coverage	30×
MIGS-30	Assemblers	Newbler version 2.5.3
MIGS-32	Gene calling method	Prodigal
	BioProject ID	PRJEB646
	Genbank Assembly ID	GCA_000403725.1
	Genbank Accession number	CAVS000000000
	Genbank Date of Release	2013/05/29
MIGS-13	Project relevance	Study of the human gut microbiome

### Growth conditions and DNA isolation

*C. ihumii* sp. nov. strain GD7^T^ strain was cultivated in Columbia broth (BioMérieux) at 37°C. Chromosomal DNA was extracted from 50mL of culture, following centrifugation at 4^o^C at 2000 xg for 20 min. Cell pellets were resuspended in 1 mL Tris/EDTA/NaCl [10mM Tris/HCl (pH7.0), 10 mM EDTA (pH8.0), and 300 mM NaCl] and re-centrifuged under the same conditions. The pellets were then re-suspended in 200µL TE buffer and proteinase K and kept overnight at 37°C for cell lysis. DNA purification with phenol/chloroform/isoamylalcohol (25:24:1) was followed by an overnight precipitation with ethanol at -20^0^C. Then, the DNA was resuspended in 200 µL TE buffer. DNA concentration was 18.3ng/µl as measured using the Genios Tecan fluorometer and the Quant-it Picogreen kit (Invitrogen).

### Genome sequencing and assembly

The 454 GS-FLX Titanium paired-end protocol (Roche, Meylan, France) was used for the library construction of *C. ihumii* strain GD7^T^ which was then pyrosequenced. Briefly, 3.7µg of purified chromosomal DNA was mechanically fragmented on the Covaris device (KBioScience-LGC Genomics, Middlesex, UK) through miniTUBE-Red with an enrichment size at 5kb. The DNA fragmentation was visualized through the Agilent 2100 BioAnalyzer on a DNA labchip 7500 with an optimal size of 2.5 kb. Circularization and nebulization were performed on 100ng of the fragmented DNA and generated an optimal pattern of 443 bp. This was followed by 17 PCR amplification cycles followed by double size selection. The single stranded paired-end library was then quantified using Quant-it Ribogreen kit (Invitrogen) on the Genios_Tecan fluorometer at 207 pg/µL. The library concentration equivalence was calculated as 8.57E+08 molecules/µL. The library was stored at -20°C until further use. The shotgun library was clonally amplified with 0.5cpb and 1cpb in 2 emPCR reactions for each condition, using the GS Titanium SV emPCR Kit (Lib-L) v2 (Roche).The yield of the shotgun emPCR reactions was 5.27 and 7.56% respectively for the two kinds of paired-end emPCR reactions according to the quality expected (range of 5 to 20%) from the Roche procedure. The library was loaded on the 1/4 region of a GS Titanium PicoTiterPlate (PTP Kit 70x75, Roche) and pyrosequenced with the GS Titanium Sequencing Kit XLR70 and the GS FLX Titanium sequencer (Roche). The run was performed overnight and analyzed on the cluster through the gsRunBrowser and Newbler assembler (Roche). A total of 186,723 passed filter wells were obtained and generated 69.4Mb with a length average of 371 bp. The passed filter sequences were assembled using Newbler with 90% identity and 40bp as overlap. The assembly lead to 5 scaffolds and 41 large contigs (>1500bp) and generated a genome size of 2,232,265 bp which corresponds to a coverage of 30.84× genome equivalent.

### Genome annotation

Open Reading Frames (ORFs) prediction was performed using Prodigal [48] with default parameters. The predicted ORFs were excluded if they spanned a sequencing gap region. Functional assessment of protein sequences was carried out by comparing them with sequences in the GenBank [49] and Clusters of Orthologus Groups (COG) databases using BLASTP. tRNAs, rRNAs, signal peptides and transmembrane helices were identified using tRNAscan-SE 1.21 [50], RNAmmer [51], SignalP [52] and TMHMM [53], respectively. ORFans were identified if their BLASTP *E-* value was lower than 1e^-3^ for alignment lengths greater than 80 amino acids. If alignment lengths were smaller than 80 amino acids, we used an *E-* value of 1e^-5^ [54]. PHAST was used to identify, annotate and graphically display prophage sequences within bacterial genomes or plasmids [55]. Artemis [56] was used for data management whereas DNA Plotter [57] was used for visualization of genomic features. In-house perl and bash scripts were used to automate these routine tasks.

To estimate the mean level of nucleotide sequence similarity at the genome level between *C. ihumii* and another 42 members of the genus *Corynebacterium*, we used the Average Genomic Identity of Orthologous gene Sequences (AGIOS) home-made pipeline. Briefly, this pipeline combines the Proteinortho software (with the following parameters: e-value 1e^-5^, 30% of identity, 50% coverage and algebraic connectivity of 50%) [58] for detecting orthologous proteins between genomes compared pairwise, then retrieves the corresponding genes and determines the mean percentage of nucleotide sequence identity among orthologous ORFs using the Needleman-Wunsch global alignment algorithm.

## Genome properties

**Figure 6:**
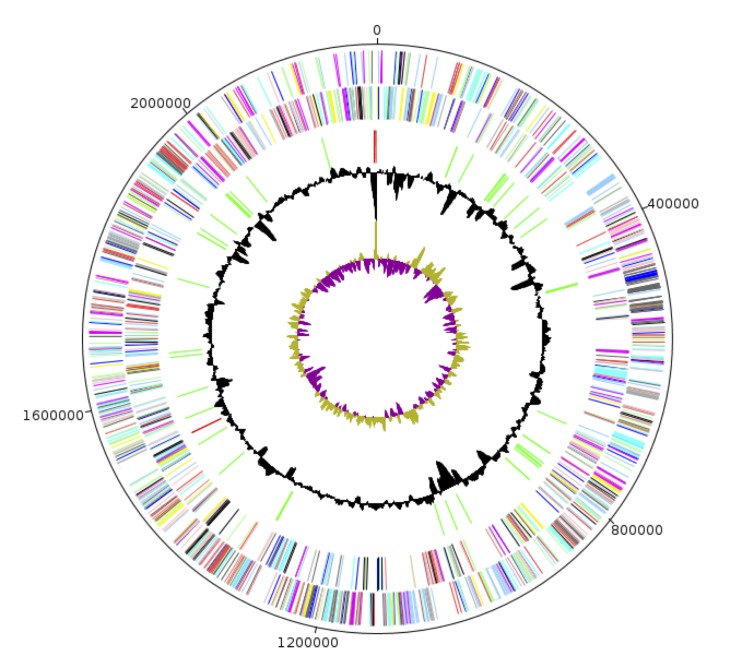
Graphical circular map of the chromosome. From the outside in, the outer two circles show open reading frames oriented in the forward and reverse directions (colored by COG categories), respectively. The third circle marks the rRNA gene operon (red) and tRNA genes (green). The fourth circle shows the G+C% content plot. The inner-most circle shows the GC skew, purple and olive indicating negative and positive values, respectively.

The genome of *C. ihumii* sp. nov. strain GD7^T^ is 2,232,265 bp long (1 chromosome in 5 scaffolds, no plasmid) with a 65.1% GC content (Table 4, Figure 6). Of the 2,182 predicted genes, 2,125 were protein-coding genes and 57 were RNAs (53 tRNA and 4 rRNA genes). A total of 1,562 genes (71.58%) were assigned a putative function. Four hundred and twenty-two genes (19.8%) were annotated as hypothetical proteins, and 126 genes ORFans (5.9%). The distribution of genes into COGs functional categories is presented in Table 5. The properties and statistics of the genome are summarized in Tables 4 and 5. A quick search with PHAST revealed that *C. ihumii* harbors an incomplete bacteriophage.

**Table 4:** Nucleotide content and gene count levels of the genome

**Attribute**	Value	% of total^a^
Genome size (bp)	2,232,265	
DNA Coding region (bp)	2,041,113	91.43
DNA G+C content (bp)	1,453,204	65.1
Number of replicons	1	
Extrachromosomal elements	0	
Total genes	2,182	100
RNA genes	57	2.61
rRNA operons	1	
Predicted tRNA pseudogenes	1	
Protein-coding genes	2,125	97.38
Genes with function prediction	1,562	71.58
Genes assigned to COGs	1,703	78.04
Genes with peptide signals	189	8.66
Genes with transmembrane helices	553	25.34
CRISPR repeats	1	

^a^ The total is based on either the size of the genome in base pairs or the total number of protein coding genes in the annotated genome

**Table 5:** Number of genes associated with the 25 general COG functional categories

**Code**	**Value**	**% of total**^a^	**Description**
J	142	6.68	Translation
A	1	0.05	RNA processing and modification
K	131	6.16	Transcription
L	114	5.36	Replication, recombination and repair
B	0	0.00	Chromatin structure and dynamics
D	19	0.89	Cell cycle control, mitosis and meiosis
Y	0	0.00	Nuclear structure
V	31	1.46	Defense mechanisms
T	60	2.82	Signal transduction mechanisms
M	95	4.47	Cell wall/membrane biogenesis
N	1	0.05	Cell motility
Z	0	0.00	Cytoskeleton
W	0	0.00	Extracellular structures
U	22	1.04	Intracellular trafficking and secretion
O	62	2.92	Posttranslational modification, protein turnover, chaperones
C	83	3.91	Energy production and conversion
G	100	4.71	Carbohydrate transport and metabolism
E	158	7.44	Amino acid transport and metabolism
F	63	2.96	Nucleotide transport and metabolism
H	78	3.67	Coenzyme transport and metabolism
I	46	2.16	Lipid transport and metabolism
P	117	5.51	Inorganic ion transport and metabolism
Q	35	1.64	Secondary metabolites biosynthesis, transport and catabolism
R	204	9.60	General function prediction only
S	141	6.63	Function unknown
-	422	19.8	Not in COGs

^a^ The total is based on the total number of protein coding genes in the annotated genome.

## Comparative genomics

Presently there are more than 75 genomic sequences (finished or draft) available for *Corynebacterium* species in GenBank. Here, we have compared *C. ihumii* sp. nov. strain GD7^T^ with 41 finished or draft genome sequences from 25 *Corynebacterium* species. Table 6 shows a comparison of genome size, GC%, coding-density, and numbers of proteins for the compared *Corynebacterium* genomes. *C. ihumii* had a smaller genome than all other compared genomes except that of *C. urealyticum* strain DSM 7111. AGIOS values identities ranged from 65.23 to 80.59% among *Corynebacterium* species, and from 97.97 to 99.99% within *Corynebacterium* species (Supplementary Table). By comparison with other species, *C. ihumii* exhibited AGIOS values ranging from 67.15% with *C. pseudotuberculosis* to 76.30% with *C. lipophiloflavum*, thus confirming its new species status.

**Table 6:** Main characteristics of *Corynebacterium* genomes compared to that of *C. ihumii* strain GD7^T^.

Species	Strain	NCIBI ID	Coding density	Length (bp)	GC%	Proteins
*Corynebacterium ihumii*	GD7^T^		90.65	2,232,265	64.95	2,125
*Corynebacterium accolens*	ATCC 49726	uid52361	86.51	2,465,976	59.23	2,360
*Corynebacterium ammoniagenes*	DSM 20306	uid48813	90.3	2,764,417	55.56	2,654
*Corynebacterium amycolatum*	SK46	uid55411	85.4	2,514,382	58.58	2,103
*Corynebacterium casei*		uid78139	85.95	3,113,786	55.34	2,700
*Corynebacterium aurimucosum*	ATCC 700975	uid59409	88.49	2,790,189	60.63	2,531
*Corynebacterium bovis*	DSM 20582	uid67345	85.72	2,527,982	72.55	2,339
*Corynebacterium diphtheriae*	VA01	uid84305	88.36	2,395,441	53.44	2,191
*Corynebacterium diphtheriae*	HC01	uid84297	88.03	2,427,149	53.43	2,248
*Corynebacterium diphtheriae*	HC02	uid84317	87.7	2,468,612	53.71	2,230
*Corynebacterium diphtheriae*	INCA 402	uid83605	87.72	2,449,071	53.65	2,214
*Corynebacterium diphtheriae*	NCTC 13129	uid57691	87.96	2,488,635	53.48	2,272
*Corynebacterium diphtheriae*	241	uid83607	87.87	2,426,551	53.43	2,245
*Corynebacterium durum*	F0235	uid183766	90.37	2,809,766	56.84	2,823
*Corynebacterium efficiens*	YS 314	uid62905	91.38	3,147,090	63.14	2,938
*Corynebacterium genitalium*	ATCC 33030	uid52785	90.81	2,349,953	62.73	2,226
*Corynebacterium glucuronolyticum*	ATCC 51867	uid55397	85.44	2,809,779	59.09	2,645
*Corynebacterium glutamicum*	R	uid58897	86.83	3,314,179	54.13	3,052
*Corynebacterium glutamicum*	ATCC 13032	uid57905	86.41	3,309,401	53.81	2,993
*Corynebacterium glutamicum*	ATCC 13032	uid61611	87.53	3,282,708	53.84	3,057
*Corynebacterium jeikeium*	K411	uid58399	89.41	2,462,499	61.4	2,104
*Corynebacterium kroppenstedtii*	DSM 44385	uid59411	86.73	2,446,804	57.46	2,018
*Corynebacterium lipophiloflavum*	DSM 44291	uid55469	87.87	2,386,544	64.26	2,371
*Corynebacterium matruchotii*	ATCC 14266	uid51885	86.43	2,856,058	57.09	2,619
*Corynebacterium nuruki*	S6 4	uid77677	89.61	3,107,265	69.49	2,797
*Corynebacterium pseudogenitalium*	ATCC 33035	uid55395	89.9	2,601,506	59.53	2,493
*Corynebacterium pseudotuberculosis*	FRC41	uid50585	87.91	2,337,913	52.19	2,110
*Corynebacterium pseudotuberculosis*	1002	uid159677	85.31	2,337,913	52.19	2,090
*Corynebacterium pseudotuberculosis*	267	uid162175	86.54	2,337,628	52.19	2,148
*Corynebacterium pseudotuberculosis*	42 02 A	uid159669	84.23	2,337,606	52.19	2,051
*Corynebacterium pseudotuberculosis*	P54B96	uid157909	84.93	2,337,657	52.19	2,084
*Corynebacterium resistens*	DSM 45100	uid50555	87.87	2,601,311	57.09	2,171
*Corynebacterium striatum*	ATCC 6940	uid55471	86.33	2,829,831	59.05	2,677
*Corynebacterium tuberculostearicum*	SK141	uid55413	89.57	2,372,621	60.01	2,210
*Corynebacterium ulcerans*	809	uid159659	87.66	2,502,095	53.3	2,180
*Corynebacterium ulcerans*	102	uid169879	87.66	2,579,188	53.36	2,349
*Corynebacterium ulcerans*	BR AD22	uid68291	87.72	2,606,374	53.4	2,334
*Corynebacterium urealyticum*	DSM 7109	uid61639	89.7	2,369,219	64.19	2,022
*Corynebacterium urealyticum*	DSM 7111	uid188688	88.17	2,316,065	64.24	1,935
*Corynebacterium variabile*	DSM 44702	uid62003	87.56	3,433,007	67.15	3,039

**Figure 7:**
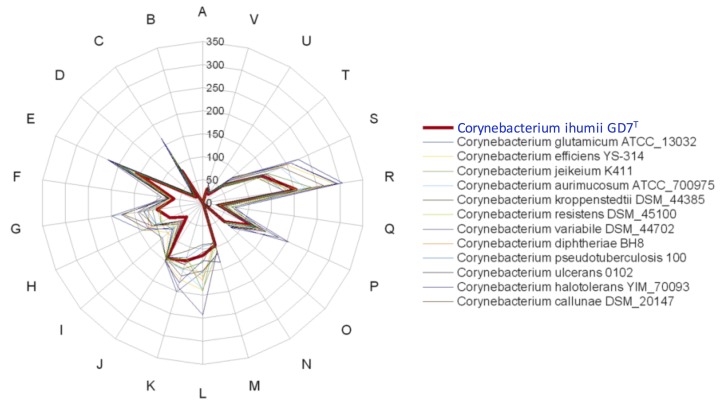
Distribution of functional classes of predicted genes of *Corynebacterium ihumii* strain GD7^T^ (colored in thick red line) along with other *Corynebacterium* genomes according to the clusters of orthologous groups of proteins.

Figure 7 shows the comparison of gene distribution into COG categories of *C. ihumii* with *C. glutamicum* strain ATCC 13032, *C. efficiens* YS 314, *C. jeikeium* K411, *C. aurimucosum* ATCC 700975, *C. kroppenstedtii* DSM 44385, *C. resistens* DSM 45100, *C. variabile* DSM 44702, *C. diphtheriae* BH8, *C. pseudotuberculosis* 1002, *C. ulcerans* 0102, *C. halotolerans* YIM 70093 and *C. callunae* DSM 20147. The overall COG distribution is similar, except *C. variabile* for category L genes.

## Conclusion

On the basis of phenotypic, phylogenetic and genomic analyses, we formally propose the creation of *Corynebacterium ihumii* sp. nov. which contains strain GD7^T^ (= CSUR P902 = DSM 45751). This bacterium was isolated from the fecal flora of a 62 year-old male admitted in intensive care unit for respiratory distress.

### Description of *Corynebacterium ihumii* strain GD7^T^ sp. nov

*Corynebacterium ihumii* (i.hum.i’i. N.L. gen. n. ihumii, based on the acronym IHUMI, the Institut Hospitalo-Universitaire Méditerranée-Infection, where the type strain was isolated). .

Colonies are white and granular with a 0.5 mm diameter on blood-enriched Columbia agar. Cells are rod-shaped with a mean length and diameter of 1.26 µm (range 1.1 – 1.4) and 0.7 µm (range 0.6-0.85), respectively. Growth is observed between 30 and 45°C, with optimal growth obtained at 37°C on blood-enriched Columbia agar. Optimal growth is achieved aerobically, but cell growth is also observed under microaerophilic and anaerobic conditions. Cells stain Gram-positive, are nonmotile and nonsporulating. Catalase is positive, oxidase is negative. Using the API ZYM system, positive reactions are observed for alkaline phosphatase, leucine arylamidase, valine arylamidase, cystin arylamidase, acid phosphatase and naphthol-AS-BI-phosphohydrolase. Negative reactions are observed for esterase (C4), esterase lipase (C8), lipase (C14), trypsin, α-chemotrypsin, α-galactosidase, β-galactosidase, β-glucuronidase, α-glucosidase, N actetyl-β-glucosaminidase, α-mannosidase and α-fucosidase. Using the API CORYNE system, positive reactions are observed for pyrazinamidase, alkaline phosphatase, and glucose and ribose fermentation. Negative reactions are observed for reduction of nitrates, pyrolidonyl arylamidase; β-glucuronidase, β-galactosidase, α-glucosidase N-acetyl-β-glucosaminidase, β-glucosidase, urease, gelatin hydrolysis, fermentation of xylose, mannitol, maltose, lactose, saccharose and glycogen. Using the API 50CH system, positive reactions are observed for fermentation of L-arabinose, D-ribose, D-xylose, methyl-βD xylopranoside, D-galactose, D-glucose, D-fructose, D-mannose, L-rhamnose, D-mannitol, methyl-αD-xylopranoside, methyl-αD-glucopranoside, N-acetylglucosamine, amygdalin, arbutin, salicin, D-cellobiose, D-maltose, D-lactose, D-mellibiose, D-saccharose, D-trehalose, inulin, D-raffinose, amidon, glycogen and D-lyxose. Negative reactions are observed for fermentation of glycerol, erythritol, D-arabinose, L-xylose, D-adonitol, L-sorbose, dulcitol, inositol, D-sorbitol, esculin ferric citrate, D-melezitose, D- xylitol, gentiobiose, D-turanose, D-tagatose, D-fucose, L-fucose, D-arabitol, L-arabitol, potassium gluconate, and potassium 2-ketogluconate. Cells are susceptible to amoxicillin, amoxicillin-clavulanic acid, ceftriaxone, imipenem, doxycycline, vancomycin, erythromycin, rifampicin, trimethoprim/sulfamethoxazole and ciprofloxacine but was resistant to metronidazole. The G+C content of the genome is 65.1%. The 16S rRNA and genome sequences are deposited in GenBank under accession numbers JX424769 and CAVS000000000, respectively.

The type strain GD7^T^ sp. nov. (= CSUR P902 = DSM 45751) was isolated from the feces of a patient admitted to intensive care in Marseille, France.
